# Dermal Lymphatic Capillaries Do Not Obey Murray's Law

**DOI:** 10.3389/fcvm.2022.840305

**Published:** 2022-04-12

**Authors:** Anne M. Talkington, Reema B. Davis, Nicholas C. Datto, Emma R. Goodwin, Laura A. Miller, Kathleen M. Caron

**Affiliations:** ^1^Program in Bioinformatics and Computational Biology, University of North Carolina at Chapel Hill, Chapel Hill, NC, United States; ^2^Department of Mathematics, University of North Carolina at Chapel Hill, Chapel Hill, NC, United States; ^3^Department of Cell Biology and Physiology, University of North Carolina at Chapel Hill, Chapel Hill, NC, United States; ^4^Department of Mathematics, University of Arizona, Tucson, AZ, United States

**Keywords:** lymphatics, Murray's Law, computational fluid dynamics, branching structure, lymph mixing

## Abstract

Lymphatic vessels serve as a major conduit for the transport of interstitial fluid, immune cells, lipids and drugs. Therefore, increased knowledge about their development and function is relevant to clinical issues ranging from chronic inflammation and edema, to cancer metastasis to targeted drug delivery. Murray's Law is a widely-applied branching rule upheld in diverse circulatory systems including leaf venation, sponge canals, and various human organs for optimal fluid transport. Considering the unique and diverse functions of lymphatic fluid transport, we specifically address the branching of developing lymphatic capillaries, and the flow of lymph through these vessels. Using an empirically-generated dataset from wild type and genetic lymphatic insufficiency mouse models we confirmed that branching blood capillaries consistently follow Murray's Law. However surprisingly, we found that the optimization law for lymphatic vessels follows a different pattern, namely a Murray's Law exponent of ~1.45. In this case, the daughter vessels are smaller relative to the parent than would be predicted by the hypothesized radius-cubed law for impermeable vessels. By implementing a computational fluid dynamics model, we further examined the extent to which the assumptions of Murray's Law were violated. We found that the flow profiles were predominantly parabolic and reasonably followed the assumptions of Murray's Law. These data suggest an alternate hypothesis for optimization of the branching structure of the lymphatic system, which may have bearing on the unique physiological functions of lymphatics compared to the blood vascular system. Thus, it may be the case that the lymphatic branching structure is optimized to enhance lymph mixing, particle exchange, or immune cell transport, which are particularly germane to the use of lymphatics as drug delivery routes.

## Introduction

The lymphatic vascular system is a major transport network, responsible for critical tasks including the trafficking and maturation of immune cells as they respond to infection and inflammation and absorbing lipids from food (1, 2). In addition to its role in healing and nutrition, the principal function of the lymphatic vascular system is to maintain tissue homeostasis by absorbing excess interstitial fluid and returning that fluid to the blood circulation. A buildup of fluid, or lymphedema, can result from improperly draining lymph. During development, this condition, known as fetal hydrops, is most often fatal (3–5). Chronic inflammation and autoimmunity are also associated with lymphatic disorders (6, 7). Thus, lymphatics govern many aspects of normal and disease physiology and can also be hijacked for the metastasis of cancer cells or for the unwanted clearance of pharmacological therapeutics (8). Likewise, there is growing interest in capitalizing on the lymphatic vasculature for the targeted delivery of therapeutics, either to evade systemic circulatory clearance, or to provide targeted delivery based on particle size (9).

The lymphatic system is comprised of a network of vessels that develop outward from a central location to the extremities (10). Both lymph flow and interstitial pressure can impact the development and morphogenesis of lymphatic vessels (11, 12). The lymphatic capillaries, which form the most distal and initial collecting structures within tissues and interstitial space, have highly-permeable endothelial junctions and a web-like branching structure that are well-suited for fluid and immune cell uptake. Lymphatic capillaries coalesce into larger lymphatic collecting vessels which are equipped with one-way valves and lymphatic muscle cells to ensure that lymph fluid maintains directed flow. Pumping in select regions of the lymphatic system, for example within the intestinal mesentery, is sufficient to maintain passive flow through the entire network. In addition, throughout the lymphatic vascular network, there exist strong extrinsic and intrinsic pumping mechanisms to support fluid propulsion. Thus, similar to the highly branched structure of veins and arteries, the lymphatic vascular system relies on a hierarchical branching pattern with a delicate balance of fluid pressures, and intrinsic and extrinsic forces, to drive unidirectional flow of lymph from capillaries to larger collectors to ultimately drain interstitial fluid to the subclavian vein (13).

Murray's Law has been shown to describe vascular branching structures in many tissue types in many organisms, including human coronary (14) and retinal arteries and veins (15), rat arterioles (14), cat pial arteries (14), blue crab arteries (16), and leaf venation (17). It describes the relationship between a “parent” vessel and the daughters it divides into. As it holds for both veins and arteries, the direction of flow has no effect on whether Murray's Law is valid. While empirically observed, Murray's Law has been derived to minimize both the volumetric cost to maintain a system and the transport cost for driving fluid (18). The law states that


(1) $$\begin{array}{r} r_{p}^{x}=\overset{N}{\underset{i}{\sum }}r_{i}^{x} \end{array}$$

or that the radius of the parent vessel r_p_ raised to some power is equal to the sum of the N radii of the daughter vessels raised to that same power (Supplementary Material A) (18). To minimize the volume related cost in the case of fully-developed laminar, Newtonian flow in impermeable vessels, x = 3. A derivation of Murray's Law is provided in Supplementary Material A. Note that for the model assumptions to hold, there must be long distances between junctions and the vessels should be of uniform diameter (14, 18).

Table 1 provides an overview of alternative Murray's Law exponents and their interpretations. A value of x = 1 would conserve the total diameter between the parent and the daughter vessels. The value x = 2 maintains cross-sectional area, thus maintaining a constant flow velocity and minimizing power spent by diffusive transport (14). When x = 4, resistance to flow is held constant (14, 21). In a turbulent regime, this total cost for transport and maintenance is minimized at x = 2.3 (21, 22). A variety of veins and arteries have been experimentally shown to have Murray's Law exponents ranging from x = 2.7–3.2. It has been shown that in physical systems, when the “parent” radius is equal to the “daughter” radii, the system has been optimized in a trade-off that maintains the minimum transport cost while maximizing advection diffusion (7).

**Table 1:** Physical interpretation of Murray's Law exponents.

**x**	**Interpretation**	**Example**
1	Total diameter conserved	Unknown
1.4–14	Minimized volume-related cost (work done) for permeable vessels	Branching in permeable tree-networks and T-junctions (19, 20)
2	(1) Constant flow velocity (cross-sectional area), (2) minimized resistance to flow, and (3) minimized power spent by diffusion	Largest arteries
2.3	Minimized transport cost in turbulent flow	Aorta and pulmonary trunk (21, 22)
2.7–3.2	Empirically observed	Observed in human arteries (14–17, 21, 23)
3	Minimized volume-related cost (work done), assumes laminar, fully developed flow	Most midsized veins and arteries
4	Constant resistance to flow (proportional to conductance)	Unknown
All radii equal	Simultaneously reduce transport cost while enhancing convective heat transfer or advection-diffusion of chemical	Man-made systems for convective heat transfer (7)

*For fully-developed, laminar flow of a Newtonian fluid, x = 3 minimizes volume-related cost. X = 1 maintains constant total diameter, x = 2 maintains total cross-sectional area, and x = 4 gives constant resistance to flow (14, 21). For permeable vessels, x may range from 1.4 to 14 exhibiting an complex relationship with permeability*.

For permeable vessels with equal daughter diameters, it has been shown that the Murray's Law exponent of x = 3 does not minimize volumetric cost, and there is complex relationship between the ideal exponent value and increasing permeability that depends upon the branching architecture. Pepe et al. (19) considered the optimal branching structure for permeable and impermeable vessels that form a T-junction where the daughter diameters are equal. If D1 is the diameter of the parent vessel and D2 is the value of each daughter diameter, then the optimal value of D2/D1 was approximately 0.8 for impermeable vessels. As the permeability increased from 0 to 10^−1^ m^2^, the optimal value of D2/D1 decreased to 0.6 such that the daughter vessels are smaller relative to the impermeable case. Solving for the Murray's Law exponent in this case results in values that range from 1.4 to 3, with lower values corresponding to more permeable vessels. On the other hand, a study by Miguel (20) that considered a porous tree-shaped network of branching vessels showed the opposite effect. The optimal D2/D1 increased to 0.952 as the permeability increased from 0 to 10^−3^, resulting in values that increase up to 14.

In this article, we measure murine dermal lymphatic capillaries and calculate the resulting Murray's Law exponents. We demonstrate that these capillaries do not follow Murray's Law for impermeable vessels where x = 3. Instead, the average exponent has a value of x = 1.45. We also measured the diameters of lymphatic vessels in calcitonin receptor-like receptor (*Calcrl*) knockout mice, which exhibit pathological hyper-permeability of lymphatics and systemic lymphatic insufficiency (4, 24). The Murray's Law exponent for this population was not significantly different from the wild type animals. To determine whether or not flow in impermeable lymphatic capillaries is nearly parabolic and comes close to following the assumptions of Murray's Law, we use computational fluid dynamics to calculate the flow profiles through experimentally measured lymphatic junction geometries. We find that flow within the vessels is laminar and nearly parabolic. This suggests that the branching structure of the lymphatic capillaries may follow an optimization rule for permeable rather than impermeable vessels. Thus, these data support the concept that the branching structure of lymphatic capillaries are uniquely different than the blood vascular system, in support of the unique physiological properties of lymphatics.

## Materials and Methods

Detailed methods are provided in Supplemental Materials B–D.

### Data Collection

Both wild type and calcitonin receptor-like receptor (*Calcrl*^*fl*/*fl*^*/CACC-CreER*^*T*2^, or herein referred to as *Calcrl*^−/−^) knockout mice (4) were considered, which represented a healthy population and a population with a leaky vessel, or lymphedema phenotype, respectively. Tissues from wild type (*n* = 16) and *Calcrl*^−/−^ knockout (*n* = 14) mice were stained for lymphatic vessel endothelial hyaluronan receptor 1 (LYVE-1), an extracellular link domain, to detect lymphatic endothelial cells and imaged with a fluorescence microscope. Tissue samples were taken from the ear, back, forelimb, hind limb, and chest. We evaluated samples from both E14.5 (embryonic day 14.5) and adult mice (3–6 months old). Wild type mice (*n* = 8) were also stained for the surface protein platelet endothelial cell adhesion molecule (PECAM) to detect blood vasculature and imaged with a fluorescence microscope.

### Murray's Law Analysis

**Figure 1:**
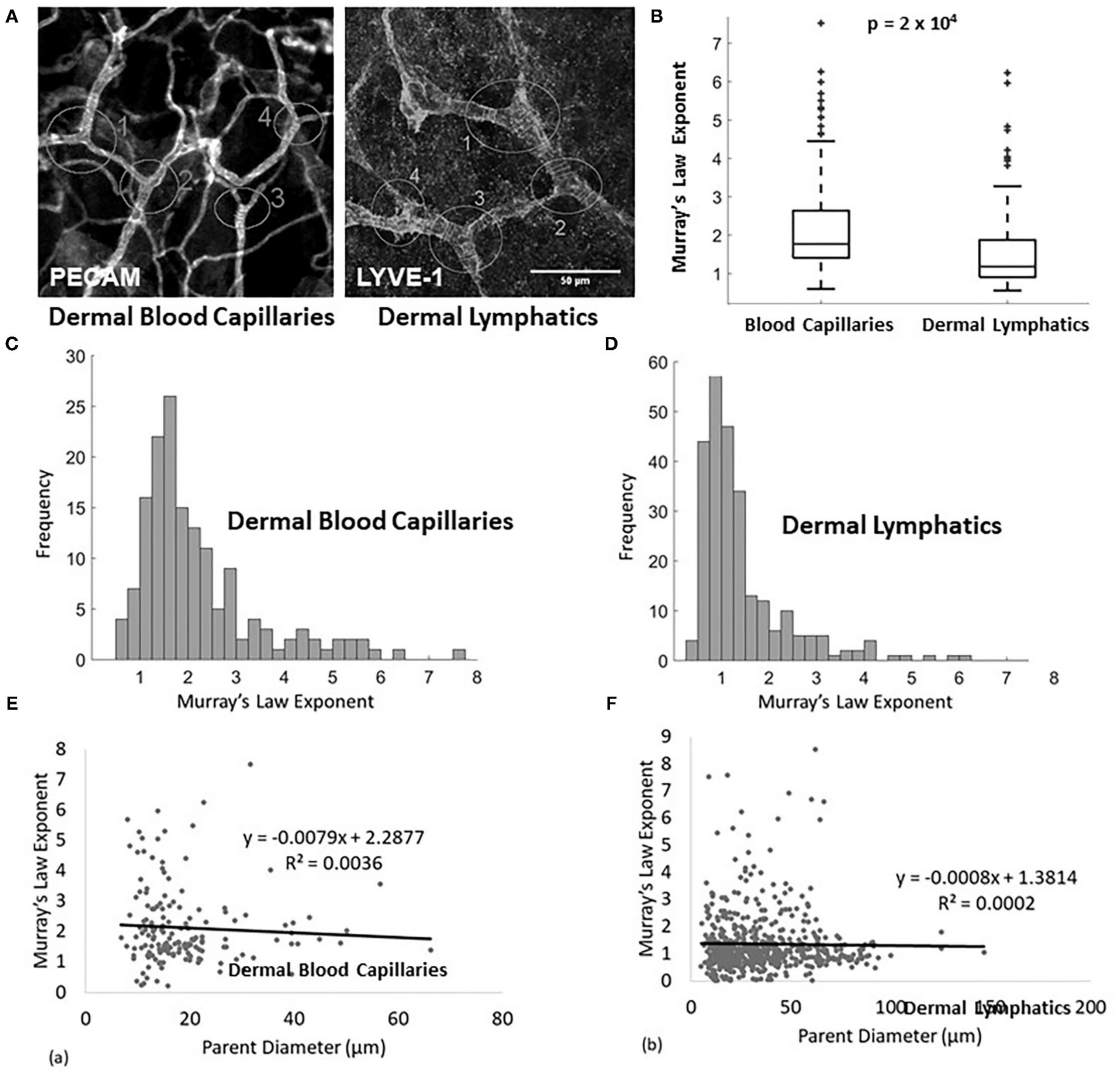
**(A)** Example of measured lymphatic (LYVE1 stain) and blood capillaries (PECAM stain) of a wild type E14.5 mouse. The analyzed junctions are identified with white circles and measured diameters are marked with white lines. **(B)** Murray's Law exponent for blood capillaries (*n* = 155 measurements, *n* = 8 mice) and lymphatic capillaries (*n* = 93 measurements, *n* = 8 mice) in wild type adult mice. **(C)** Murray's Law exponent distribution for blood capillaries. The right skew is a result of variability in a sample with a mean close to and bounded by 0. **(D)** Murray's Law exponent distribution for dermal lymphatic capillaries in wild type mice. The right skew is a result of variability in a sample with a mean close to and bounded by 0. **(E,F)** The calculated Murray's Law exponents do not correlate with parent vessel size at the scale of the measured capillaries for **(E)** blood or **(F)** dermal lymphatics. The exponent distributions are not skewed due to size effects.

The fluorescence microscopy images were contrast-adjusted as needed to obtain clear vessel boundaries. Junctions were identified for Murray's Law analysis, where a “parent” vessel branched into its “daughter” vessels (Figure 1A). A parent vessel was defined as the largest branch with the restriction that the branching angle between the daughters must be smaller than the branching angle between each daughter and the parent. At each junction, three diameter measurements were taken along each vessel and averaged. Diameters were measured at 3 representative points along each vessel corresponding to, but not immediately adjacent to, each bifurcation point. The image processing was completed in ImageJ (25, 26). The average values were then used to calculate a Murray's Law exponent for each junction by solving equation 1.1. A total of 531 lymphatic measurements and 155 blood vessel measurements, excluding exponent values >10 and <0.35, were obtained. The distribution of these exponents was then analyzed. Two-tailed *t*-tests were applied to determine the significance of tissue type and *Calcrl* genotype. A one-tailed *t*-test was applied to compare the dermal lymphatic capillary sample with the blood capillary sample. Welch's correction was applied to account for unequal variance, and statistical differences in variance were analyzed with an *F*-test. To validate the assumption that the vessels are approximately cylindrical, a series of z-stack images were obtained from E14.5 dermal lymphatic capillaries. These capillaries were then visualized and the dimensions quantified in ImageJ. On average, the measured axes were within 20%, a difference of <10 microns, of each other. It is reasonable to treat these capillaries as cylindrical when filled with fluid (27).

### Numerical Experimental Design

Five lymphatic junctions with Murray's Law exponents of approximately 1 were reconstructed using MeshmerizeMe, a meshing software designed to identify the boundary of the lymphatic vessel and export the boundary as a set of discrete vertex points (28). Two larger lymphatic networks were also similarly reconstructed and discretized. Fluid was simulated through these networks using the immersed boundary method (29, 30). See Supplemental Material C for detailed methods.

## Results

### Characterizing the Murray's Law Exponent Distribution

The Murray's Law exponent distribution for murine dermal lymphatic capillaries has a mean of approximately 1 (1.440 ± 0.064) (mean ± standard error), with the peak of the right-tailed distribution occurring at x ≈ 1 (Figures 1B,D). This indicates that the daughters are smaller than would be expected relative to the parent, had they upheld the x = 3 rule derived to minimize transport cost. In fact, they come closer to following a strictly additive rule (x = 1). No significant differences are observed when the sample is stratified by tissue type. The Murray's Law exponents associated with dermal lymphatic capillaries are significantly smaller than the exponents in blood capillaries measured in the same tissues (*p* = 2 × 10^−4^) (Figures 1B,C). These blood capillaries follow a Murray's Law exponent distribution with a mean of approximately 2.2 (2.208 ± 0.101) (mean ± standard error), which in itself is significantly smaller (*p* < 10^−4^) than the x = 3 observed in larger veins and arteries (Figure 1C) but still within a reasonable range (21). Figures 1E,F show the calculated Murray's Law exponent for blood capillaries (Figure 1E) and dermal lymphatic capillaries (Figure 1F). Note that the blood capillaries and vessels range in size from about 5–70 microns and the dermal lymphatic capillaries range in size from about 10 to 150 microns. In both cases, there is no significant correlation between parent diameter and the Murray's Law exponent. This suggests that non-Newtonian effects, which can be of significance at the smallest scale, do not significantly affect the Murray's Law exponent.

**Figure 2:**
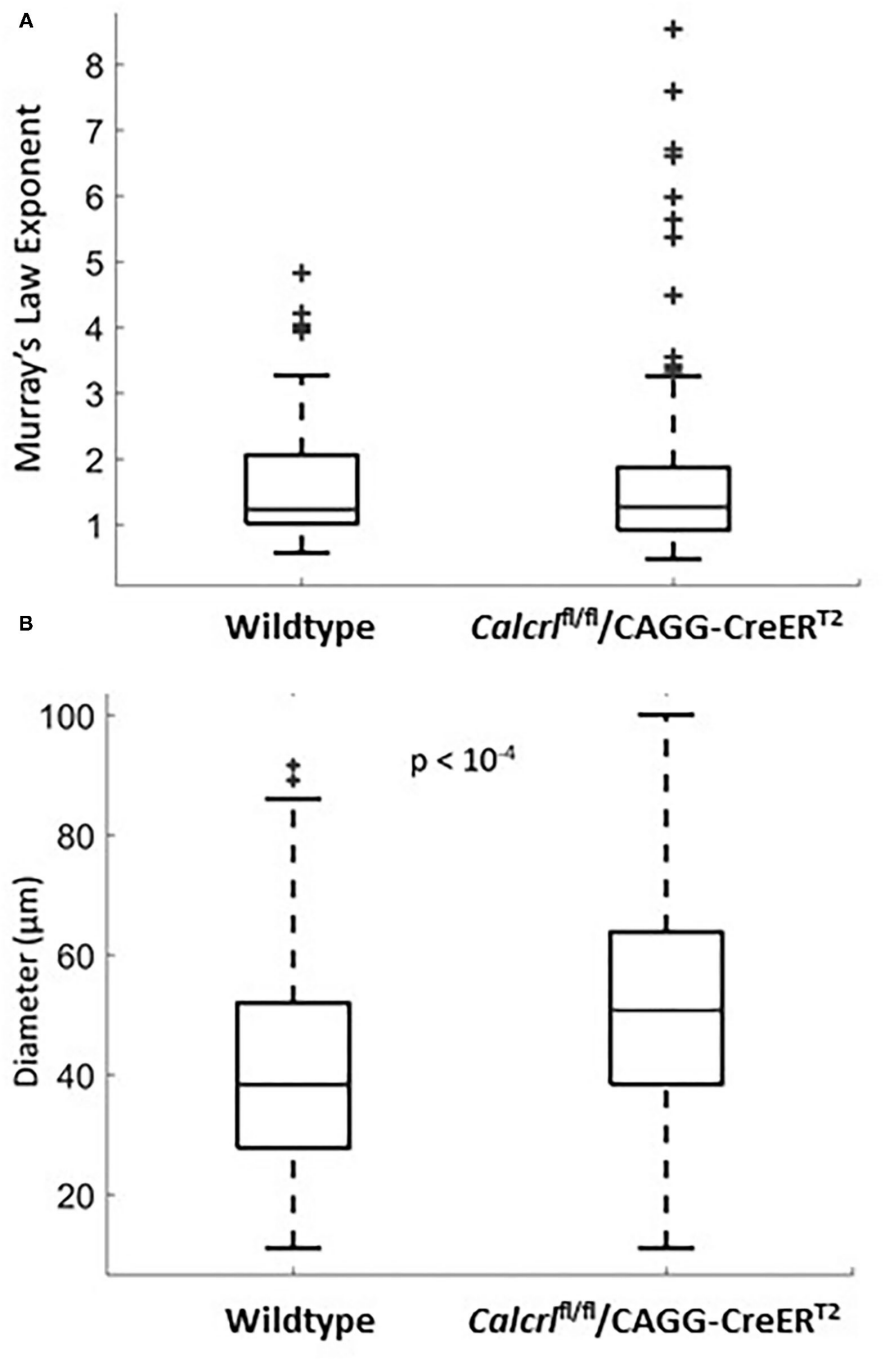
**(A)** Murray's Law exponent distribution for dermal lymphatic capillaries in wild type (*n* = 47 measurements, *n* = 5 mice) and *Calcrl*^−/−^ knockout (*n* = 131 measurements, *n* = 8 mice). All measurements were taken in adult ear tissue. Note the extent of the spread in the *Calcrl*^−/−^ distribution. **(B)** Diameter distribution for dermal lymphatic capillaries in wild type (*n* = 115 measurements, *n* = 8 mice) and *Calcrl*^−/−^ knockout (*n* = 143 measurements, *n* = 9 mice) adult mice.

To determine whether pathological changes in lymphatic permeability or systemic lymphatic insufficiency could influence the branching pattern of dermal lymphatic capillaries, we evaluated Murray's Law exponents in dermal lymphatics from adult mice globally deficient for the G protein-coupled receptor, calcitonin receptor-like receptor, which as a receptor for adrenomedullin and CGRP peptides, represents an essential signaling pathway for lymphatic development and function (4, 24). Compared to the wild type mice, the *Calcrl*^−/−^ knockout mice are not significantly different with regard to their mean exponent (Figure 2A). However, the variance of the Murray's Law exponent in the *Calcrl*^−/−^ knockout population is significantly greater than that of the wild type (*p* = 0.044 <0.05), which is consistent with previous reports of functional lymphatic insufficiency in this model (Figure 2A). Furthermore, we find that the mean diameter of dermal lymphatic capillaries in the *Calcrl*^−/−^ knockout population (52.1 ± 1.5 microns) is significantly larger than wild type dermal capillary diameter (41.8 ± 1.8 microns) (*p* < 10^−4^) (Figure 2B). This is also consistent with results previously observed in mesenteric vessels, and supportive of its use as a genetic model of lymphatic hyperpermeability and lymphedema (2). Thus, while there is evidence of dilated lymphatic vessels in the *Calcrl*^−/−^ knockout mice, it does not change the Murray's Law exponent of dermal lymphatic capillaries.

### Computational Fluids Modeling

**Figure 3:**
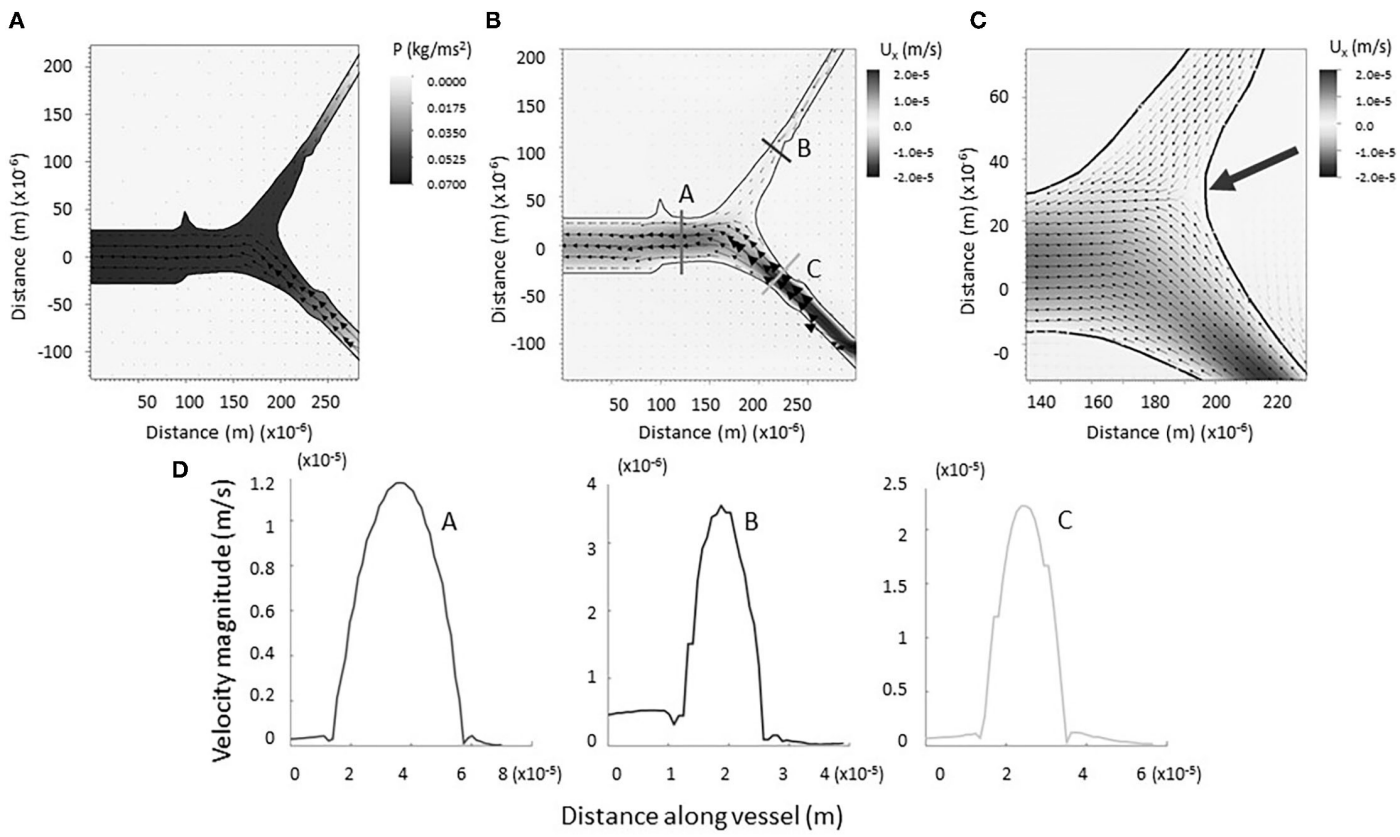
**(A)** Computational model of lymph flowing through x = 1 junction. Note the formation of a negative pressure gradient (heat map) driving the flow of lymph as it is drawn into the vessel. Vectors represent fluid velocity. **(B)** Computational model of lymph flowing through x = 1 junction. No regions of backflow are evident. Vectors represent fluid velocity, and the heat-scale map shows the horizontal component of the flow. **(C)** Bifurcation of the vessels is marked by a stagnation point (large arrow). **(D)** Magnitude of flow velocity taken along cross sections shown in B. This verifies that flow is nearly parabolic within the vessels (coded by lettering).

The physical impact of vessel geometry on the flow of lymph through the system can be examined through a computational fluid model using an actual lymphatic junction from our data (Figure 3A). Figures 3B,C shows a heat map of the horizontal component of the velocity, and the arrows point in the direction of flow. Figure 3D shows the magnitude of the velocity taken along cross-sections of the vessel, as indicated in panel B. For the initial computational modeling, the numerical simulation was constructed for a dermal lymphatic capillary junction in a wild type mouse assuming that the vessel wall is impermeable and nearly rigid. Initial visualization of the resolved flow field is qualitatively consistent with bifurcating channel flow. The smaller daughter takes up fluid at a slower velocity due to increased resistance. The larger daughter takes up fluid at a higher velocity, as the fluid is pulled into the structure. The range of velocities maintains its consistency with biologically observed lymphatic flow velocity, as low as 10^−7^ and as high as 10^−3^m/s (31, 32). The arrow in Figure 3C shows a stagnation point at the junction. A close examination of the velocity vectors within the vessels show no areas of flow reversal, and nearly parabolic flow is preserved. This pattern was observed in all four junctions that were simulated (see Supplementary Materials E.3–6).

As expected, a negative pressure gradient develops as the lymph flows out of the parent vessel, presumably into a larger network that drains into a collecting vessel (see Figure 3A). This draws the lymph into the primary vessels and junction from the surrounding region, modeling the biological process of lymphatic fluid regulation in the body. Reported values correspond to velocities resulting from the prescribed velocity boundary conditions. The small magnitudes, up to the order of 10^−2^ N/m^2^, are consistent with the knowledge that dermal lymphatic capillaries operate in a low-pressure environment (33).

**Figure 4:**
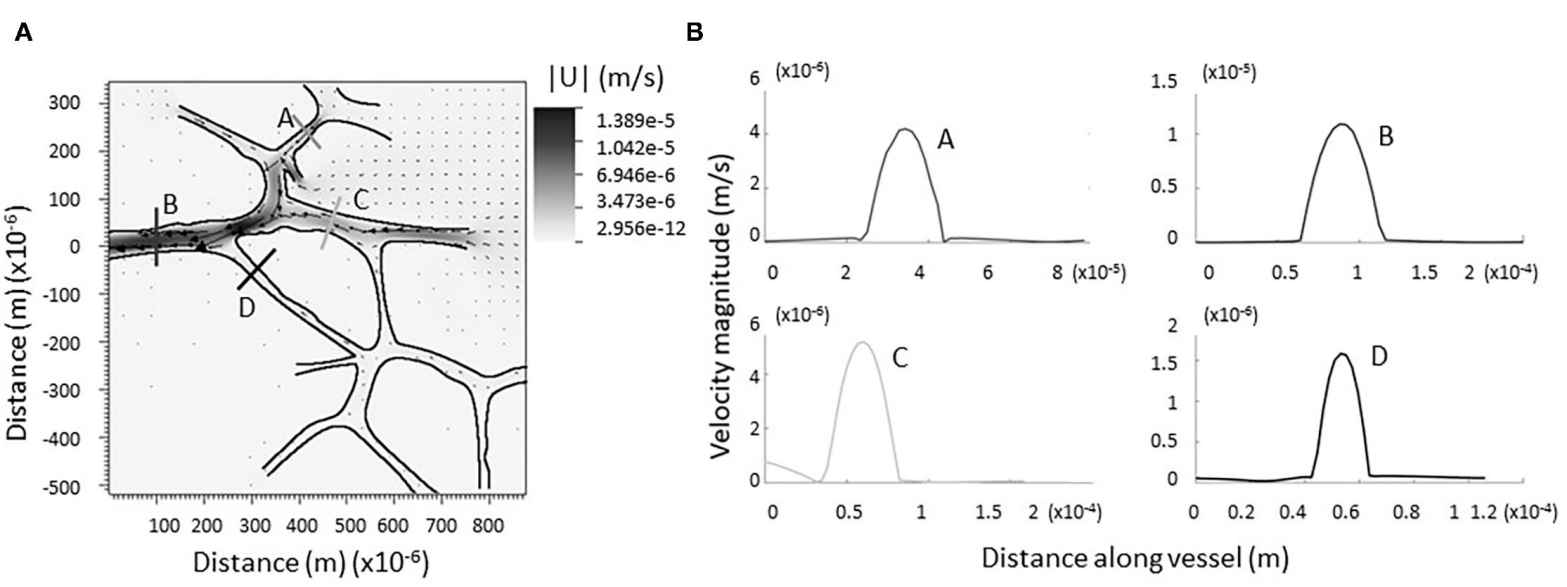
Computational model of lymph flowing through larger network of dermal capillaries. **(A)** As in the single junction analysis, no regions of backflow are evident. The trend is preserved in each individual junction. Vectors represent the direction and magnitude of flow. The heat-scale map gives the magnitude of the velocity. **(B)** Verification flow remains parabolic or nearly parabolic at representative cross sections (coded by lettering).

Figure 4A shows a grayscale heatmap of the magnitude of the velocity as well as velocity vectors describing the flow through a network of 9 junctions. Figure 4B shows the magnitude of the velocity taken along four cross-sections in the network. Note that the flow in all cases is nearly parabolic and no regions of flow reversal are immediately observed. A similar pattern is found for another network provided in Supplementary Figure E.7. There are notable stagnation points at the branching regions associated with new capillary formation (see Figures 3B,C, Supplementary Figure E.8). This observation is consistent with the idea that changes in fluid flow, sensed by biological mechanosensors for either stagnation or laminar shear, can signal lymphangiogenesis.

We also measured the tortuosity of the lymphatic capillaries as a proxy for uneven shear along the vessel wall. Tortuosity was measured as the actual vessel length between two bifurcation points divided by the shortest distance between the branches. Tortuosity values >1 imply that surface area is increased in this system. We found tortuosity values for dermal lymphatic capillaries to have a mean of 1.07 ± 0.04. Higher values of tortuosity result in increased surface area which could allow for greater fluid and immune cell exchange between the vasculature and the interstitium. This potential increase in exchange is consistent with reported values for enhanced exchange of 5% in blood vessels of comparable size (34). Additionally, although deviation from fully developed flow was modest, vessel tortuosity implies uneven shear along the wall, which would violate an assumption of Murray's Law.

## Discussion

Using an empirical dataset that we generated from wild type and genetic models, we demonstrate that murine dermal lymphatic capillaries do not follow Murray's Law for x = 3. In other words, the lymphatic network does not follow a minimization rule for volumetric cost that assumes impermeable vessels with fully developed, laminar flow. The best fit mean exponent is approximately 1.45. This is within the range of a volumetric cost minimization rule for permeable T-junctions. We note that lymphatic capillaries are permeable, and Murray's Law exponents for optimal transport in permeable vessels will differ from the optimal value of 3 for impermeable vessels. This may be particularly relevant to larger lymphatic vessels, such as pre-collectors ad collectors, which have lymphatic smooth muscle cells and tighter cellular junctions. Indeed, the physiological functions and regulation of these larger, less permeable lymphatics differs significantly from the dermal lymphatics modeled in this study, such that Murray's Law exponents for pre-collectors and collectors might be predicted to differ as well.

Unfortunately, the permeability of dermal lymphatic vessels has not been well characterized, and it is not currently possible to determine whether or not the branching structure is optimized accordingly. However, considering the unique fluid absorptive and mixing properties of dermal lymphatics, it is also likely that the branching structure is representative of an advantageous design for fluid uptake, lymph mixing or immune cell trafficking. The later hypotheses would require an optimization rule outside of Murray's Law for ideal fluid transport. The derivation of such a rule would need to account for other features of vascular structure and function, such as the vessels' ability to dilate, exchange chemicals, traffic large immune cells and respond to bother intrinsic and extrinsic pumping forces (35, 36).

We also found that relative to wild type mice, *Calcrl*^−/−^ mice have a similar mean exponent but with greater variation. These mice display hyperpermeable lymphatic vessels which are insufficient for maintaining lymphatic transport in numerous organs (2). Therefore, the increased variation in the Murray's Law exponent could be a result of increased permeability and lack or dysfunction of structural proteins in the knockout mice, consistent with their lymphedema phenotype.

In addition to efficient lymph transport, it is necessary for the structure of the lymphatic capillaries to ensure well-mixed fluid in the daughter vessels. As the lymph is pulled upstream into the network, mixing is critical for avoiding a buildup of chemokines, cytokines, or other inflammatory agents and chemicals, like drugs and nanoparticles. While mixing ability in the daughters may appear to be an important feature of lymphatic structure, computational fluid simulations using impermeable vessels suggests that this is not necessarily the case. On the other hand, the observed increased surface area of permeable vessels suggests that immune cell transport may play a greater role in the optimized function of dermal lymphatic capillaries. This is supported by the calculated tortuosities which are representative of increased transport in analogous blood capillaries (34). It is also worth considering that vessels of this size scale are subject to non-Newtonian effects and shear thinning, which could suggest a different optimization strategy for fluid maintenance or transport, as demonstrated by blood capillaries (37). There has, however, been evidence that conservation of fluid transport has led to preservation of Murray's Law in even tiny capillaries (21, 23).

Future work and extensions of the numerical model include the incorporation of elasticity or porosity to account for vessel permeability, as well as the incorporation of spatiotemporal regulators of extrinsic and intrinsic forces on lymphatic capillaries (35, 36). The ability of permeable walls to affect flow properties and hence diameter ratios has been suggested (19, 36). However, the conservation of Murray's Law in some porous systems suggests that the problem of vascular geometry is more complex than simply minimizing fluid transport cost (17). Developing an appropriate optimization rule is even more challenging if permeability varies in time and space, as is the case for lymphatic capillaries (38). Furthermore, at higher Reynolds numbers in controlled T-structures, permeability has been shown to affect the symmetry of flow (19). Changes in the porosity of the system affect properties such as the resistance of the fluid as it moves through the structure and its tendency to form a stagnation point at a bifurcating junction, here relevant for signaling lymphangiogenesis (19). An expanded model, run over longer times and accounting for a larger network, could capture these additional properties to address alternative hypotheses in greater detail. Indeed, measurement of intrinsic pumping of rat dermal lymphatics reveals temporally elongated contraction-relation cycles and continuously varying flow and backflow (38). Thus, as the simulation increases in complexity, it holds the potential to model the flow (or lack thereof) in lymphedema, parameterized with the *Calcrl*^−/−^ mouse model, or a gradient buildup in the case of targeted drug delivery via lymphatic routes.

## Data Availability Statement

The raw data supporting the conclusions of this article will be made available by the authors, without undue reservation.

## Ethics Statement

The animal study was reviewed and approved by Institutional Animal Care and Use Committee of UNC-CH.

## Author Contributions

AT designed the study, collected data, performed experiments, analyzed data, and wrote the manuscript. RD prepared animal tissues for the study, advised on study design, and supervised data analysis. ND and EG collected and analyzed data. LM and KC conceived the research hypothesis, designed the study, analyzed data, supervised data analysis and interpretation, edited the manuscript, and provided funding for the study. All authors contributed to the article and approved the submitted version.

## Funding

This work was supported in part by grants from the National Institutes of Health, NHLBI R01 HL129086 and NIDDK R01 DK119145 to KC and the National Science Foundation IOS #1558052 and DMS #1151478. This material was based upon work supported by the National Science Foundation Graduate Research Fellowship Program under Grant No. DGE-1650116.

## Author Disclaimer

Any opinions, findings, and conclusions or recommendations expressed in this material are those of the authors and do not necessarily reflect the views of the National Science Foundation.

## Conflict of Interest

The authors declare that the research was conducted in the absence of any commercial or financial relationships that could be construed as a potential conflict of interest.

## Publisher's Note

All claims expressed in this article are solely those of the authors and do not necessarily represent those of their affiliated organizations, or those of the publisher, the editors and the reviewers. Any product that may be evaluated in this article, or claim that may be made by its manufacturer, is not guaranteed or endorsed by the publisher.
