# Supplementary material for: Dermal Lymphatic Capillaries Do Not Obey Murray's Law
Source: Front Cardiovasc Med. 2022 Apr 12;9:840305. doi: 10.3389/fcvm.2022.840305 (PMC9039365; doi:10.3389/fcvm.2022.840305)
Supplement: Supplementary file 1 [file Data_Sheet_1.PDF]

Dermal lymphatic capillaries do not obey Murray's Law - Supplementary Materials

A Derivation of Murray's Law

Murray's Law has been derived to minimize transport cost in a branching structure assuming laminar flow. We accomplish this by minimizing the total power needed for fluid transport in a laminar flow regime. The total power is the sum of the transport power P_t and maintenance power P_m. Given that

$$P_t = \frac{8\mu l}{\pi r^4} Q^2 \tag{1}$$

where μ is dynamic viscosity, l is the length of the transporting vessel, r is vessel radius, and Q is laminar flow rate; given Poiseuille flow; and given

$$P_m = \lambda \pi l r^2 \tag{2}$$

where λ is a constant metabolic factor, and maintenance cost is proportional to the volume of the vessel, we arrive at our objective function

$$P = P_t + P_m = \frac{8\mu l}{\pi r^4} Q^2 + \lambda \pi l r^2 \tag{3}$$

We then optimize power as follows:

$$\frac{dP}{dr} = 0 = -\frac{32\mu l}{\pi r^5} Q^2 + 2\lambda \pi l r \tag{4}$$

Rearranging, we can solve for flow rate Q as a function of radius r:

$$\frac{32\mu l}{\pi r^5} Q^2 = 2\lambda \pi l r \tag{5}$$

which implies

$$Q = \frac{\pi}{4} \sqrt{\frac{\lambda}{\mu}} r^3 \tag{6}$$

We arrive at a function relating the cubed radius to the laminar flow rate. Summing the flow in the daughter vessels Q_i, we arrive at

$$Q_p = \sum_{i=1}^{n} Q_i \implies \frac{\pi}{4} \sqrt{\frac{\lambda}{\mu}} r_p^3 = \sum_{i=1}^{n} \frac{\pi}{4} \sqrt{\frac{\lambda}{\mu}} r_i^3 \tag{7}$$

Dividing our proportionality constant, we find the familiar form of Murray's Law (Equation 1.1), where $x = 3$:

$$r_p^3 = \sum_i r_i^3$$

B Detailed Experimental Methods

B.1 Animals

Animal procedures used in this study were approved by the University of North Carolina Chapel Hill's Institutional Animal Care and Use Committee. *Calcrl*$^{fl/fl}$, *Calcrl*$^{fl/fl}$/*CAGG-CreER*^{T2}, and *Calcrl*$^{fl/fl}$/*Prox1-CreER*^{T2} adult animals used in this study were generated and genotyped as per *Hoopes et al., 2012*[36] and *Davis et al., 2017*[2]. All mixed background *Calcrl*$^{fl/fl}$ and *Calcrl*$^{fl/fl}$/*Prox1-CreER*^{T2} embryos were generated by crossing *Calcrl* females to *Calcrl*$^{fl/fl}$/*Prox1-CreER*^{T2} adult males. *Calcrl* was excised by Cre-mediated recombination initiated by tamoxifen (Sigma Aldrich T5648 - dissolved in corn oil and ethanol) administration for 5 days for adults and 3 days for E8.5 to E10.5 in pregnant dams for embryos at a dose of 5 mg/40 g intraperitoneally. Embryonic back skin was harvested at E14.5. Adult ear and chest skin was harvested from *Calcrl*$^{fl/fl}$/*CAGG-CreER*^{T2} animals 1 year after tamoxifen and from *Calcrl*$^{fl/fl}$/*CAGG-CreER*^{T2} animals 2 weeks after tamoxifen for further processing.

B.2 Tissue Processing and Immunofluorescence

Mouse embryos were fixed in 4% paraformaldehyde in PBS for overnight at 4 °C. Back and limb skin was collected for staining as per *Li et al., 2011.* Briefly, skins were peeled upon dehydration in 100% MeOH followed by rehydration staining. Whole mount skin was washed with PBS and permeabilized in 0.1% Triton X-100 in PBS, blocked for 1 hour at room temperature in 5% normal donkey serum in PBS. Primary antibodies for rabbit anti-LYVE1 (1:250, Fitzgerald) and rat anti-PECAM (1:100, BD Pharmingen 550274) were used overnight at 4 °C. After washing with 0.1% Triton X-100 in PBS samples were probed with donkey anti-rabbit Cy2 (1:200, 711-225-152 Jackson ImmunoResearch) and donkey anti-rat Cy3 (1:400, 712-165-150, Jackson ImmunoResearch) conjugated secondary antibodies for 1.5 hours at room temperature in the dark. After washing with PBS samples were mounted on slides in Prolong gold mounting media (Life technologies, P36934), coverslipped and imaged.

Adult mouse skin samples were obtained from the back and the ears. Briefly, hair was removed from the back skin using Nair and then a small section cut and fixed on filter paper in 2%PFA at 4C overnight. Skin from the ears were similarly fixed followed by peeling and staining the external layer. Staining procedure was performed similarly across embryonic and adult skin tissues.

B.3 Microscopy and Image Analysis

Whole mount fluorescently stained skin was imaged on either a Nikon E800 fluorescence microscope with a Hamamatsu Orca camera and MetaMorph software (Molecular Devices Corporation) or a Zeiss LSM 700 Confocal Laser scanning microscope and ZEN 2011 software. All images were psuedocolored and analyzed using ImageJ (NIH) and Fiji.

C Detailed Numerical Methods

The immersed boundary method was used to solve the fluid-structure interaction problem of an incompressible, Newtonian fluid (lymph) flowing within vessels[28]. In particular, the Immersed Boundary Method with Adaptive Mesh Refinement (IBAMR) software library was used for this study[28,37,38]. As a first approximation for this initial study, the boundary was assumed to be nearly rigid and impermeable, and the structure was 2-dimensional. To compensate for the boundary conditions of open-ended vessels, the ends of the vessels were artificially extended to create a parabolic flow profile.

The fluid domain was specified as a square with length 5×10^{-4}m. The Lagrangian vertex points describing the vessel walls were spaced approximately $L/2048$ units apart, where L is the size of the fluid domain. These boundary points were held in place by tethering them to fixed target points. The Navier-Stokes equations were solved on a Cartesian grid where the finest level of refinement has a spatial discretization of $dx = L/1024$. Note that the distance between the boundary points ds was specified as half the distance between the Cartesian points dx. The lymphatic fluid was parameterized according to previously published results, with density $\rho = 1000$ kg/m^3 and dynamic viscosity $\mu = 10^{-3}$ Ns/m^2, which results in a Reynolds number on the order of 10^{-3} [29]. Three levels of mesh refinement were used, with a refinement ratio of 4 between levels. Parabolic outflow was prescribed at a velocity of 10^{-5}m/s at the domain-bordering edge of the parent vessel, and temporally developed from rest according to a hyperbolic tangent function. The flow direction was specified for the lymphatic vessel as "pulling" the fluid from the daughter to the parent vessels, as opposed to "pushing" the fluid into the daughter vessels. Biologically, this corresponds to a fluid uptake process, where an upstream valve prohibits backflow. Dirichlet boundary conditions were imposed directly above and below the parent vessel and on the top and bottom walls of the domain ($u_x = u_y = 0$). The domain wall directly across from the junction was specified with the Neumann boundary condition $\frac{\partial u_x}{\partial t} = \frac{\partial u_y}{\partial t} = 0$ to allow for steady inflow, maintaining fluid conservation. The simulations were run for 1 second with $dt = 5 \times 10^{-6}$s.

As a check on the selection of the spatial grid size, we performed a numerical simulation on a network of junctions as shown in Figure 4, setting $dx = L/1024$ and $L/2048$. Figure C.1a shows the magnitude of flow taken along a cross section of the smallest vessel. The absolute error along that distance is shown

in Figure C.1b. This represents the worst case scenario, and in most cases the error was less than $\approx 2\%$. Even in this worst case, peak velocities and the overall velocity profiles are quite similar. Additional support for this resolution can be found in Figures E.1 and E.2.

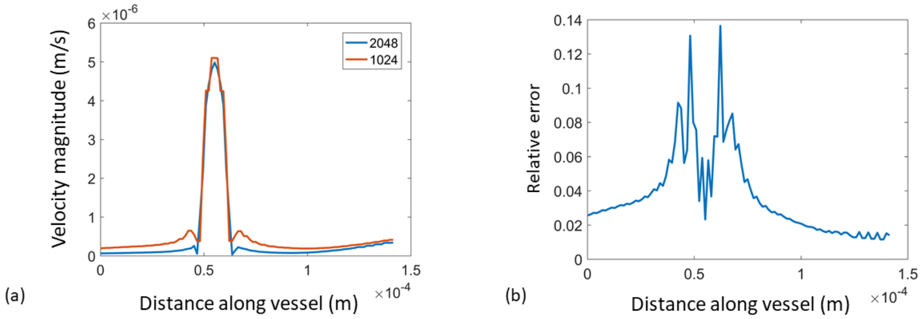

Figure C.1: Convergence study for the smallest vessel in the lymphatic network. The spatial discretization was set to $dx = L/1024$ and $L/2048$. (a) The velocity along the cross section, and (b) the relative error in velocity. The maximum error occurred near the vessel boundary, and the peak velocities were quite similar.

D The Immersed Boundary Method

The Immersed Boundary (IB) method is a mathematical formulation and a numerical method for solving the fully-coupled fluid-structure interaction problems of an elastic structure immersed in a viscous fluid[28]. The Navier-Stokes equations describing a viscous incompressible fluid are given as

$$\rho(\mathbf{u}_t(\mathbf{x},t) + \mathbf{u}(\mathbf{x},t) \cdot \nabla \mathbf{u}(\mathbf{x},t)) = -\nabla p(\mathbf{x},t) + \mu \nabla^2 \mathbf{u}(\mathbf{x},t) + \mathbf{f}(\mathbf{x},t), \quad (8)$$

$$\nabla \cdot \mathbf{u}(\mathbf{x},t) = 0, \quad (9)$$

where $\mathbf{x}$ represents spatial position in Cartesian coordinates, t represents time, $\mathbf{u}$ represents the velocity of the fluid, p represents pressure, $\mathbf{f}$ represents the force per unit area applied to the fluid by the boundary, and μ represents dynamic

viscosity. The interaction equations between the fluid and the elastic boundary are expressed as integral equations with delta function kernels and are given as

$$\mathbf{f}(\mathbf{x},t) = \int \mathbf{F}(q,t)\delta(\mathbf{x} - \mathbf{X}(q,t))dq, \tag{10}$$

$$\mathbf{X}_t(q,t) = \mathbf{U}(\mathbf{X}(q,t)) = \int \mathbf{u}(\mathbf{x},t), \delta(\mathbf{x} - \mathbf{X}(q,t))d\mathbf{x} \tag{11}$$

where q represents the position along the curvilinear boundary, $\mathbf{X}_t(q,t)$ gives the Cartesian coordinates of the boundary at position q, and $\mathbf{F}(q,t)$ is the force per unit length along the boundary, and $\mathbf{X}_t(q,t) = \mathbf{U}(\mathbf{X}(q,t))$ is the velocity of the boundary at the position q. Note that Equation 11 enforces the no-slip condition, and Equation 10 spreads the elastic force on the boundary to the fluid. Both of these equations involves a two-dimensional Dirac delta function, δ.

The force equations are specific to the application. In a simple case where the boundary motion is prescribed or fixed, boundary points are tethered to target points. The equation describing the force applied to the fluid by the boundary in Lagrangian coordinates is given by

$$\mathbf{F}(q,t) = k_{targ}(\mathbf{Y}(q,t) - \mathbf{X}(q,t)), \tag{12}$$

where k_{targ} is a stiffness coefficient and $\mathbf{Y}(q,t)$ is the preferred position.

The Immersed Boundary method with Adaptive Mesh Refinement (IBAMR) was used to numerically solve this system of equations[37,38]. This software library uses adaptive meshing to resolve flow near the elastic boundaries and in regions of high vorticity.

E Supplementary Figures

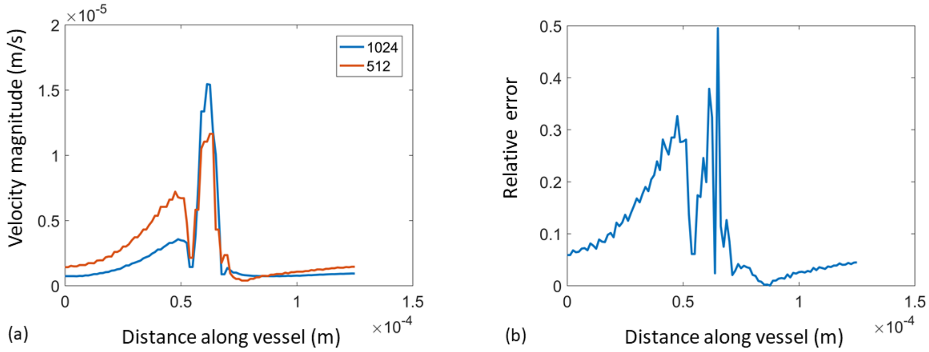

Figure E.1: (a) Convergence of the numerical model used in the tiniest vessel of figure E.7. The parabolic flow profile is illustrated for the first cross-section, highlighted in blue. 512×512 is too coarse for this structure and thus leaky; the peak is not fully captured. We see that increasing the resolution to 1024×1024 addresses leakage. (b) Although absolute error comparing the models is an order of magnitude less than the peak flow velocity, we incur large relative error. We observe convergence of the model and further support for using 1024×1024 as the optimal resolution for the full network structure.

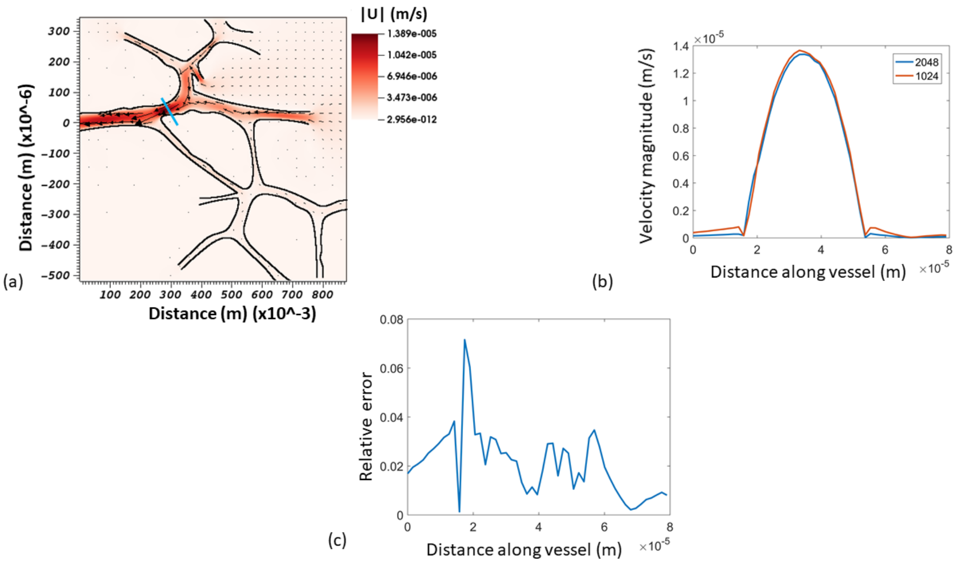

Figure E.2: Typical convergence of the numerical model, run on a 1024 × 1024 and 2048 × 2048 grid. (a) Cross-section specified for analysis, (b) the velocity along the cross section, and (c) the relative error in velocity. Note that the relative error is consistently approximately 2%.

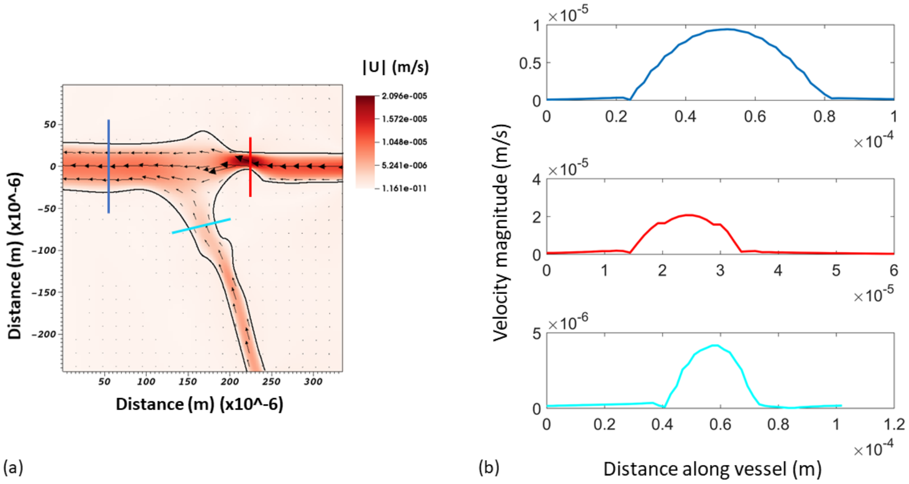

Figure E.3: (a) Flow velocity in an individual junction. (b) Flow profiles corresponding to color-coded cross sections demonstrate that the flow is nearly parabolic.

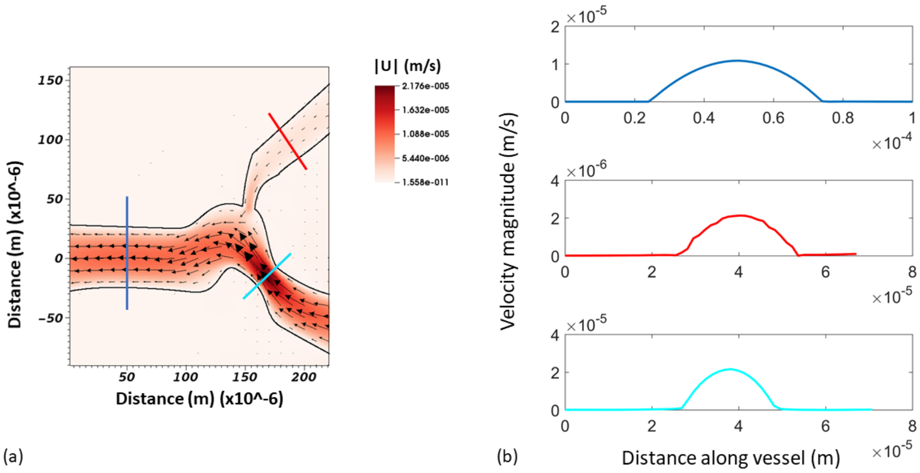

Figure E.4: (a) Flow velocity in an individual junction. (b) Flow profiles corresponding to color-coded cross sections demonstrate that the flow is nearly parabolic.

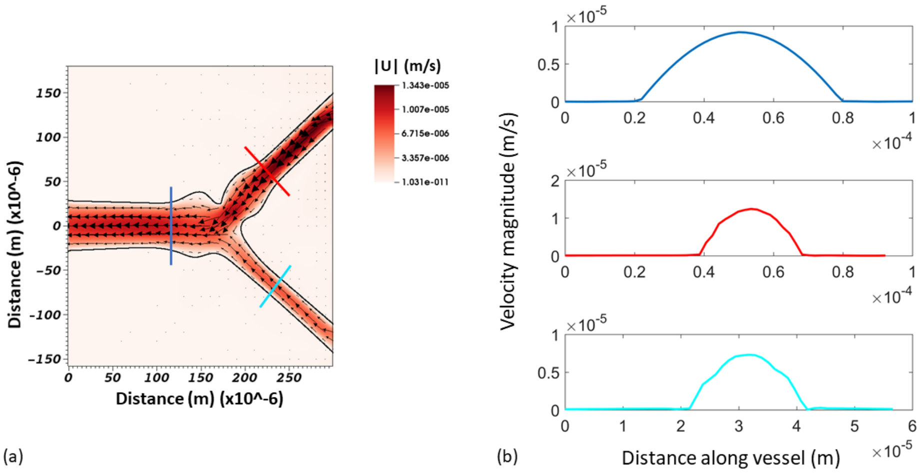

Figure E.5: (a) Flow velocity in an individual junction. (b) Flow profiles corresponding to color-coded cross sections demonstrate that the flow is nearly parabolic.

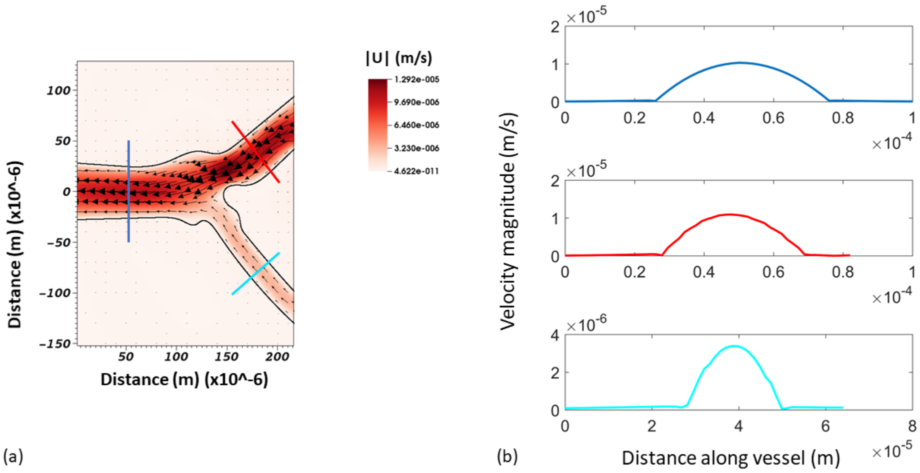

Figure E.6: (a) Flow velocity in an individual junction. (b) Flow profiles corresponding to color-coded cross sections demonstrate that the flow is nearly parabolic.

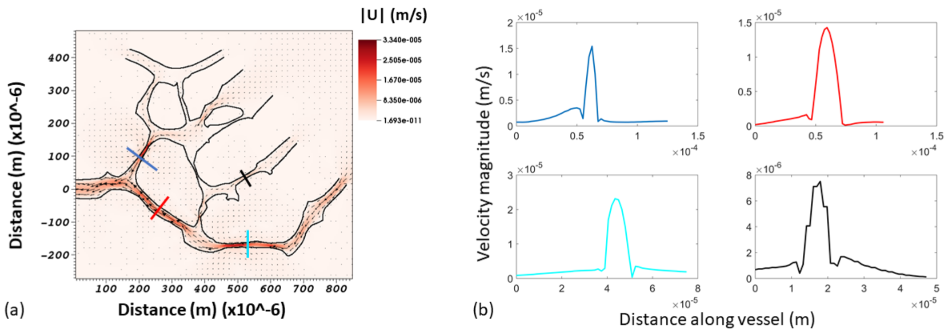

Figure E.7: (a) Flow velocity in a larger network structure on a 1024 × 1024 grid. (b) Flow profiles corresponding to color-coded cross sections demonstrate that the flow is nearly parabolic.

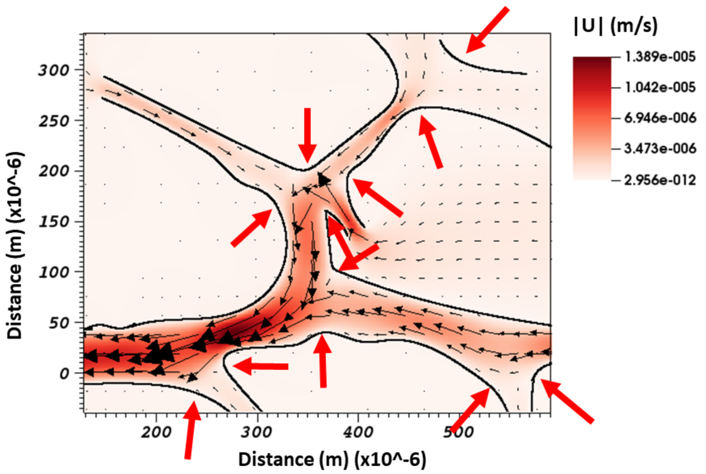

Figure E.8: Bifurcation points are characterized by local flow disturbance (red arrows).
